# Semiconductor Quantum Wells and Nanostructures

**DOI:** 10.3390/nano13131924

**Published:** 2023-06-24

**Authors:** Ze Don Kvon

**Affiliations:** 1 Rzhanov Institute of Semiconductor Physics, 630090 Novosibirsk, Russia; kvon@isp.nsc.ru; 2 Department of Physics, Novosibirsk State University, 630090 Novosibirsk, Russia

Semiconductor quantum wells and nanostructures have been the main quantum and classical physical objects in condensed matter physics for over half a century, since the discovery of the two-dimensional electron gas in silicon MOSFETs and size quantization in thin bismuth films. During this period, a large number of fundamentally novel quantum phenomena have been found in these systems, including intersubband optical transitions, ballistic conductance quantization, the Aharonov–Bohm effect in ballistic quantum rings, the Kondo effect in quantum dots, superlattice effects and Bloch oscillations, and two- and one-dimensional Anderson localization, just to name a few. However, the most significant discoveries in the field of semiconductor quantum well systems were integral and fractional quantum Hall effects. These discoveries represent the most remarkable achievements in physics during the second half of the 20th century and the first two decades of the 21st century. Following these findings, quantum mechanics obtained a clear, simple, and at the same time, fundamental signature—the conductance quantum e^2^/h. Notably, the most precise value of the fine structure constant e^2^/ħc is determined by the IQHE experiment. It is necessary to add that one of the most significant discoveries in the 21st century, microwave-induced magnetoresistance oscillations, was made in a high-mobility two-dimensional electron gas in a GaAs quantum well. Furthermore, it is worth noting that the field of semiconductor (quantum) wells physics has led to the development of a wide range of new electronic and optical devices, including HEMT, quantum cascade lasers, and quantum sensors on the basis of intersubband optical transitions.

This Special Issue of *Nanomaterials* is devoted to the latest news in the physics of Semiconductor Quantum Wells and Nanostructures connected with different aspects of contemporary condense matter physics. First of all, it is important to highlight a notable review [1] concerning recent advances in the understanding of plasma phenomena in 2D electron systems. Two papers [2,3] are devoted to the study of transport and optical properties of 2D Dirac semimetal, realized in HgTe quantum wells with a critical thickness corresponding to the transition from a direct to an inverted band structure due to relativistic effects. In [2], transport in the quantum Hall effect regime is studied in a 2D Dirac fermions system with a single Dirac cone for the first time. In [3], direct experimental evidence of the existence of an almost ideal linear spectrum in gapless HgTe QW has been obtained. There is also a related paper [4], which reports the observation of interface superconductivity in a hybrid junction of normal metal (Au) and Dirac semimetal (NiTe_2_). The coherence properties of a single heavy-hole spin qubit on the basis of a double GaAs/AlGaAs quantum dot are studied in a captivating paper [5]. An entire series of articles [6,7,8,9,10] is presented by the group of prof. C. Duque from University of Antioquia (Columbia). It clearly demonstrates that computer modeling of the dispersion and optical response of semiconductor quantum wells and nanostructures provides a wide field for such kind of activity. Another paper [11] reports on the use of quantum wells as part of a complicated device structure. In this structure, a novel concept for spin-polarized electron emission/injection combining the optocoupler principle based on a vacuum spin-polarized light-emitting diode (spin VLED) has been introduced, making it possible to measure the free electron beam polarization injected into the III-V heterostructure with quantum wells. Finally, two papers [12,13] describe different methods of fabricating semiconductor-based micro- and nanostructures. The first one describes the technology that introduces the modulated potential by means of a light beam. The second paper presents a method for the fabrication of nanowires, nanosheets, and nanorods using thermally dewetted Au nanoparticles as a catalyst in a chemical vapor deposition process.
